# Publisher Correction: Population-based heteropolymer design to mimic protein mixtures

**DOI:** 10.1038/s41586-023-06554-y

**Published:** 2023-08-30

**Authors:** Zhiyuan Ruan, Shuni Li, Alexandra Grigoropoulos, Hossein Amiri, Shayna L. Hilburg, Haotian Chen, Ivan Jayapurna, Tao Jiang, Zhaoyi Gu, Alfredo Alexander-Katz, Carlos Bustamante, Haiyan Huang, Ting Xu

**Affiliations:** 1 Department of Materials Science and Engineering, University of California Berkeley, Berkeley, CA USA; 2 Department of Statistics, University of California Berkeley, Berkeley, CA USA; 3 Institute for Quantitative Biosciences-QB3, University of California, Berkeley, CA USA; 4 Department of Materials Science and Engineering, Massachusetts Institute of Technology, Cambridge, MA USA; 5 Department of Molecular and Cell Biology, University of California Berkeley, Berkeley, CA USA; 6 Department of Chemistry, University of California Berkeley, Berkeley, CA USA; 7 Department of Physics, University of California Berkeley, Berkeley, CA USA; 8 Howard Hughes Medical Institute, University of California Berkeley, Berkeley, CA USA; 9 Center for Computational Biology, University of California, Berkeley, CA USA; 10 Materials Science Division, Lawrence Berkeley National Laboratory, Berkeley, CA USA; 11 Present Address: Department of Chemistry, Xiamen University and The MOE Key Laboratory of Spectrochemical Analysis and Instrumentation, Xiamen, China; 12 Present Address: Departments of Chemistry and Biomedical Engineering, Northwestern University, Evanston, IL USA

**Keywords:** Polymers, Fluids

Correction to: *Nature* 10.1038/s41586-022-05675-0 Published 8 March 2023

This paper was originally published under a standard Springer Nature license (© The Author(s), under exclusive licence to Springer Nature Limited). It is now available as an open-access paper under a Creative Commons Attribution 4.0 International license, © The Author(s). The error has been corrected in the HTML and PDF versions of the article.

