# Supplementary material for: Population-based heteropolymer design to mimic protein mixtures
Source: Nature. 2023 Mar 8;615(7951):251–8. doi: 10.1038/s41586-022-05675-0 (PMC10468399; doi:10.1038/s41586-022-05675-0)
Supplement: Supplementary file 1 — This file contains Supplementary Tables 1 and 2 and Figs. 1–21. [file 41586_2022_5675_MOESM1_ESM.pdf]

nature portfolio

https://doi.org/10.1038/s41586-022-05675-0

## Supplementary information

# Population-based heteropolymer design to mimic protein mixtures

In the format provided by the authors and unedited

**Supplemental Information for**

# Population-based Heteropolymer Design to Mimic Protein Mixtures

Zhiyuan Ruan[1], Shuni Li[2], Alexandra Grigoropoulos[1], Hossein Amiri[3], Shayna Hilburg[4], Haotian Chen[1], Ivan Jayapurna[1], Tao Jiang[1]‡, Zhaoyi Gu[1]†, Alfredo Alexander-Katz[4], Carlos Bustamante[3,5,6,7,8,9,10], Haiyan Huang[2], Ting Xu*[1,7,10,11,12]

**This file includes:**
Table S1-S2
Figure S1-S21

**List of Tables**

**Table S1** The conversion from amino acids to RHP monomers (N=2) ........................................................................4
**Table S2** The conversion from amino acids to RHP monomers (N=8) ........................................................................5

**List of Figures**

**Fig. S1** The length distribution of hydrophobic block for different proteins. ................................................................6
**Fig. S2** The length distribution of hydrophobic block for RHP1-7 ensembles. ............................................................7
**Fig. S3** The segment distribution of RHP ensembles and proteins in PCA space.........................................................8
**Fig. S4** The segment distribution of each RHP ensemble and proteins in PCA space (N=8). .......................................9
**Fig. S5** The segment distribution of each RHP ensemble and proteins using 2, 4, and 8 pseudo-residues. ................10
**Fig. S6** The segment distribution of each RHP ensemble as a function of the number of segments (N) per RHP ensemble. ..............................................................................................................................................12
**Fig. S7** The correlation between the relative abundance of different segments and their distribution in PCA space..13
**Fig. S8** All FEC traces upon pulling (red) and relaxing (orange) showing no unfolding signature............................18
**Fig. S9** All FEC traces upon pulling (red) and relaxing (orange) showing smooth shoulders....................................19
**Fig. S10** All FEC traces upon pulling (red) and relaxing (orange) showing discrete rips. .........................................20
**Fig. S11** All FEC traces upon pulling (red) and relaxing (orange) showing a mixture of rips and shoulders. ............21
**Fig. S12** AqpZ-eGFP fluorescence in the presence/absence of RHP ensembles during cell-free expression. ............22
**Fig. S13** OmpT-eGFP fluorescence in the absence of RHP ensembles during cell-free expression (n=3). ................23
**Fig. S14** The solvent accessible surface area (SASA) of a representative RHP4 particle over 100 ns of MD simulation.........................................................................................................................................24
**Fig. S15** The solvent accessible surface area (SASA) of each type of monomer toward the hexane phase over 100 ns of MD simulation. ...........................................................................................................................25
**Fig. S16** The solvent accessible surface area (SASA) of each type of monomer toward the water phase over 100 ns of MD simulation. ...........................................................................................................................26
**Fig. S17** DLS measurement of DHP1 polymer solution. ............................................................................27
**Fig. S18** DLS measurement of DHP2 polymer solution. ............................................................................28
**Fig. S19** DLS measurement of RHP4 polymer solution..............................................................................29
**Fig. S20** The segment distribution of RHP1, RHP14 and Proteinase K in PCA space...............................................30
**Fig. S21** 8% PAGE analysis showing the formation of DNA-RHP conjugates using thiol-maleimide addition. .......31

**Table S1** The conversion from amino acids to RHP monomers (N=2)

| ID | Hydrophobicity | Amino acid | Monomer equivalent |
|---|---|---|---|
| 1 | Hydrophobic | Leu, Ile, Phe, Trp, Val, Met, Cys, Tyr, Ala, Thr, and Gly | EHMA |
| 2 | Hydrophilic | Ser, Gln, His, Asn, Pro, Glu, Asp, Arg, and Lys | OEGMA |

**Table S2** The conversion from amino acids to RHP monomers (N=8)

| ID | Hydrophobicity | Amino acid | Monomer equivalent |
|---|---|---|---|
| 1 | Very hydrophobic 2 | Leu, Trp, Ille, Phe | EHMA |
| 2 | Very hydrophobic 1 | Met and Val | HMA[a] |
| 3 | Hydrophobic 2 | Tyr, Cys, Ala | BMA[b] |
| 4 | Hydrophobic 1 | Thr, Gly | MMA |
| 5 | Hydrophilic 2 | His, Ser and Gln | OEGMA (Mn=300 Da) |
| 6 | Hydrophilic 1 | Asn and Pro | OEGMA (Mn=500 Da) |
| 7 | Positively Charged | Arg and Lys | DMAEMA |
| 8 | Negatively Charged | Glu and Asp | SPMA |

[a] Hexyl methacrylate.

[b] Butyl methacrylate.

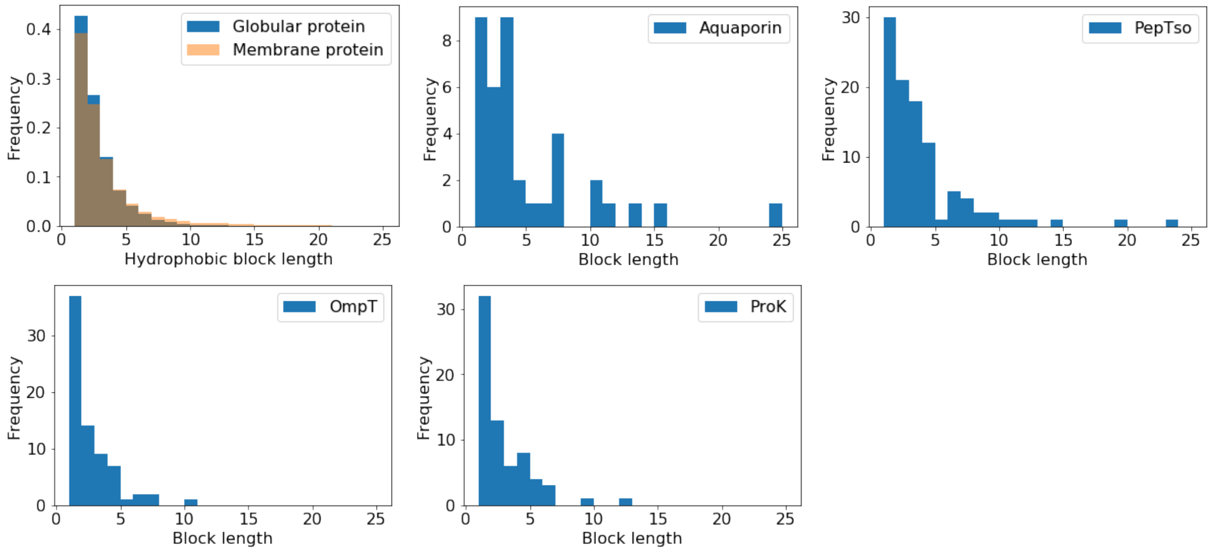


**Fig. S1** The length distribution of hydrophobic block for different proteins.

A hydrophobic block is a conseceutive motif in which each monomer is hydrophobic. Binary assignment (Table S2) was used to map 20 amino acids to 2 pseudo monomers, hydrophobic or hydrophilic. The distribution of hydrophobic block length of membrane proteins has a slightly longer tail than that of globular proteins. Individual proteins show different length distribution of hydrophobic block.

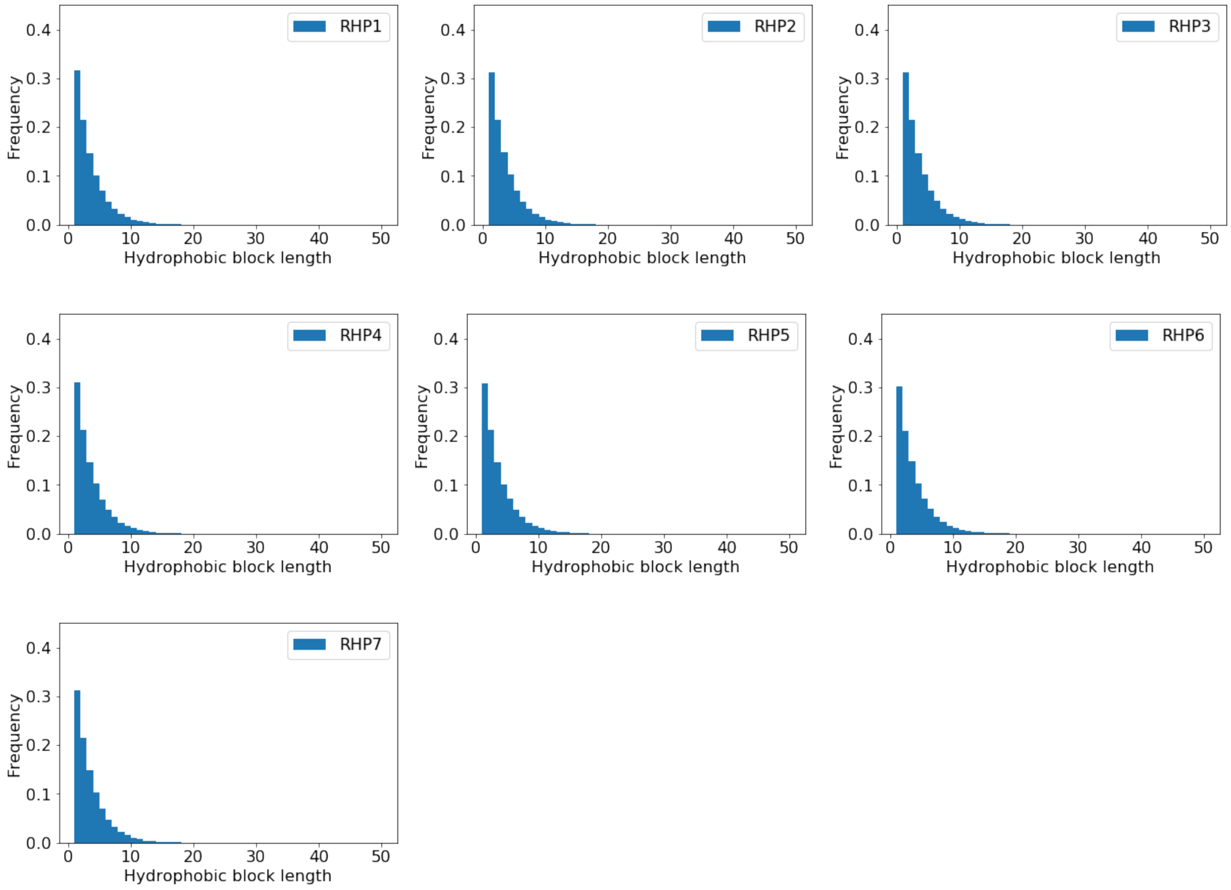


**Fig. S2** The length distribution of hydrophobic block for RHP1-7 ensembles.

MMA and EHMA are assigned as "hydrophobic", SPMA and OEGMA are assigned as "hydrophilic". A hydrophobic block is a conseceutive motif, in which each monomer is hydrophobic. This sequence analysis cannot distinguish RHP1-7 ensembles.

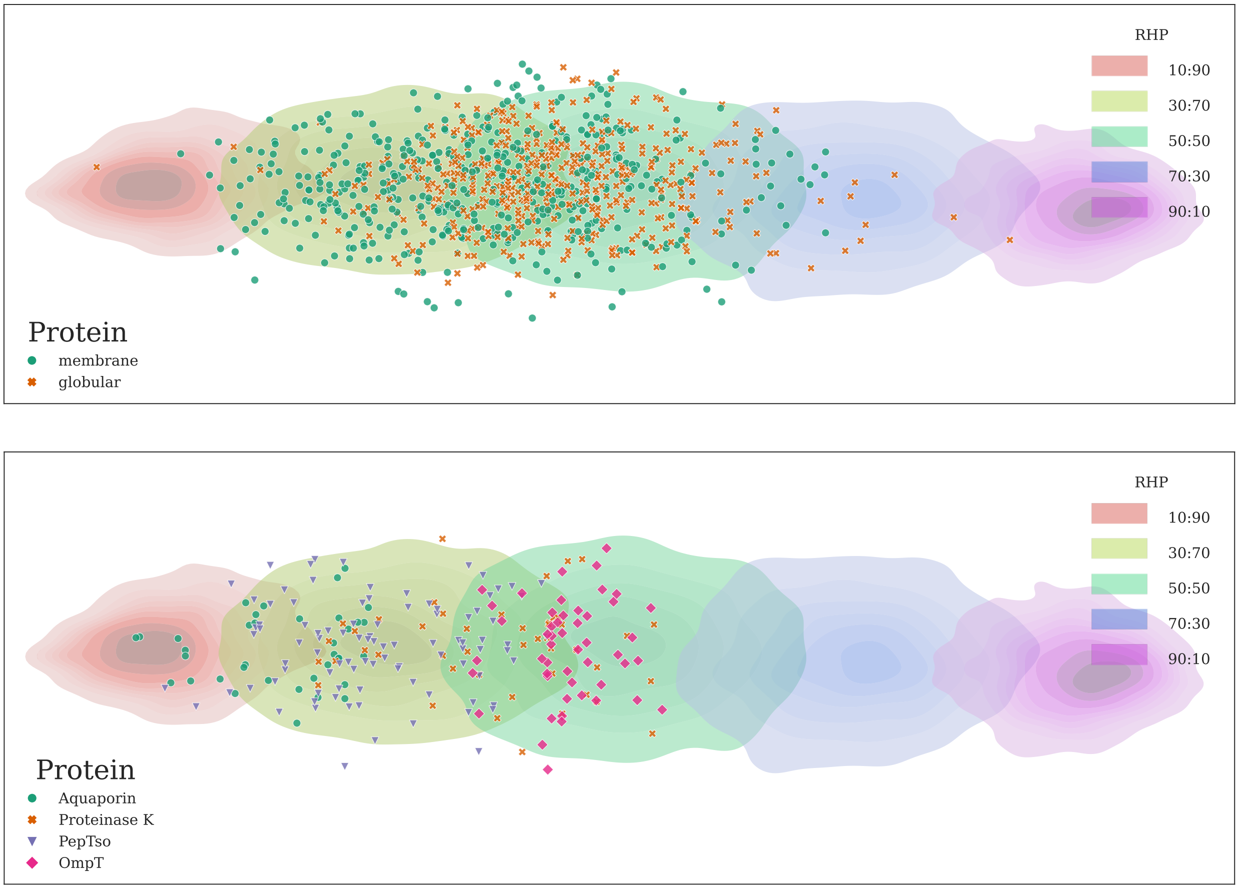


**Fig. S3** The segment distribution of RHP ensembles and proteins in PCA space.

Both sequences are encoded using 2 pseudo-residues as shown in Table S2. RHP ensembles including 30:70 and 50:50 (hydrophilic:hydrophobic) have the most overlap with both globular proteins and membrane proteins. Each RHP ensemble has a much larger sequence space than that of a single protein sequence, suggesting that a larger alphabet size is required to narrow the segment space of an RHP ensemble.

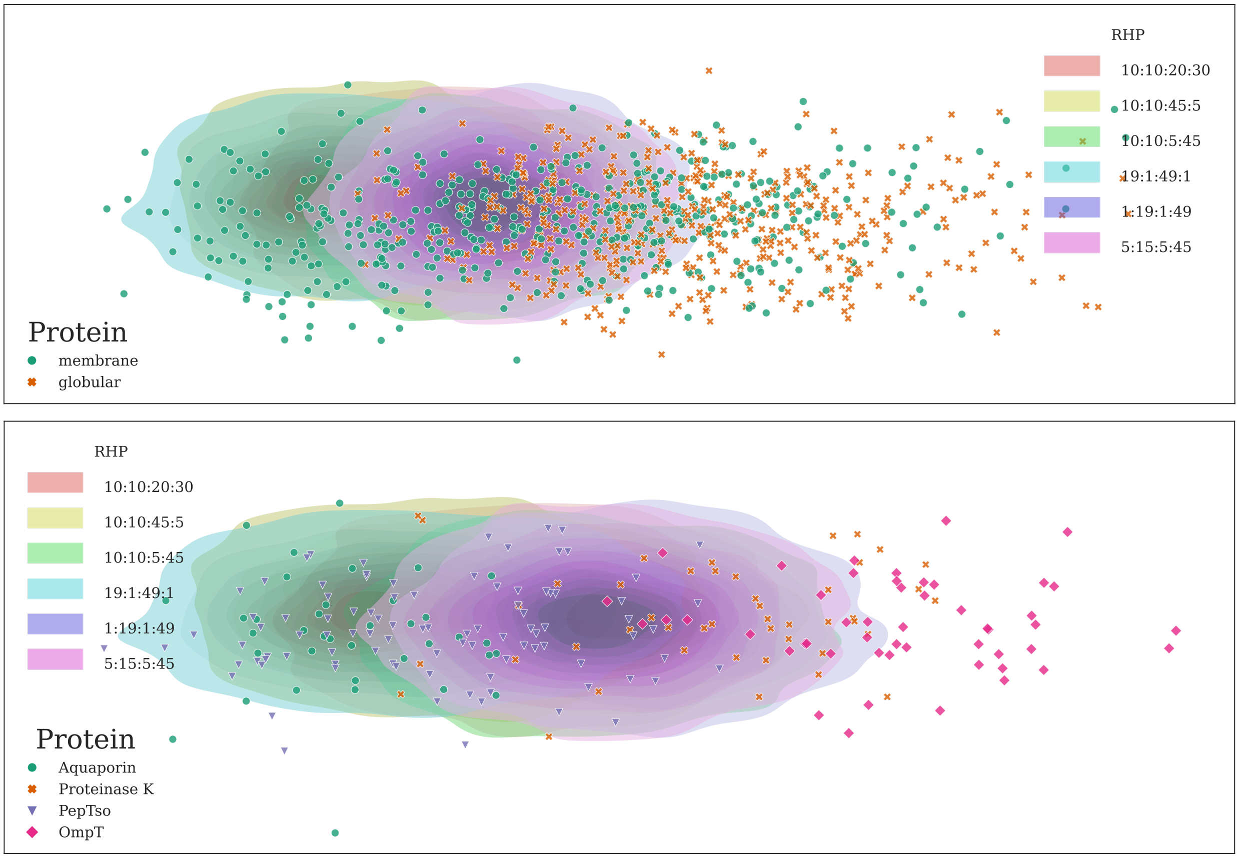


**Fig. S4** The segment distribution of each RHP ensemble and proteins in PCA space (N=8).

As the molar ratio of hydrophobic/hydrophilic (70/30) is fixed, each composition occupies a narrower sequence space than that of ensembles made of 2 monomers. 10_10_20_30 represents the ratio of 10:10:20:30:12.5:12.5:2.5:2.5 for monomer 1-8 as listed in Table S3. The composition of hydrophilic residues remains the same as (12.5:12.5:2.5:2.5) for all RHP ensembles.

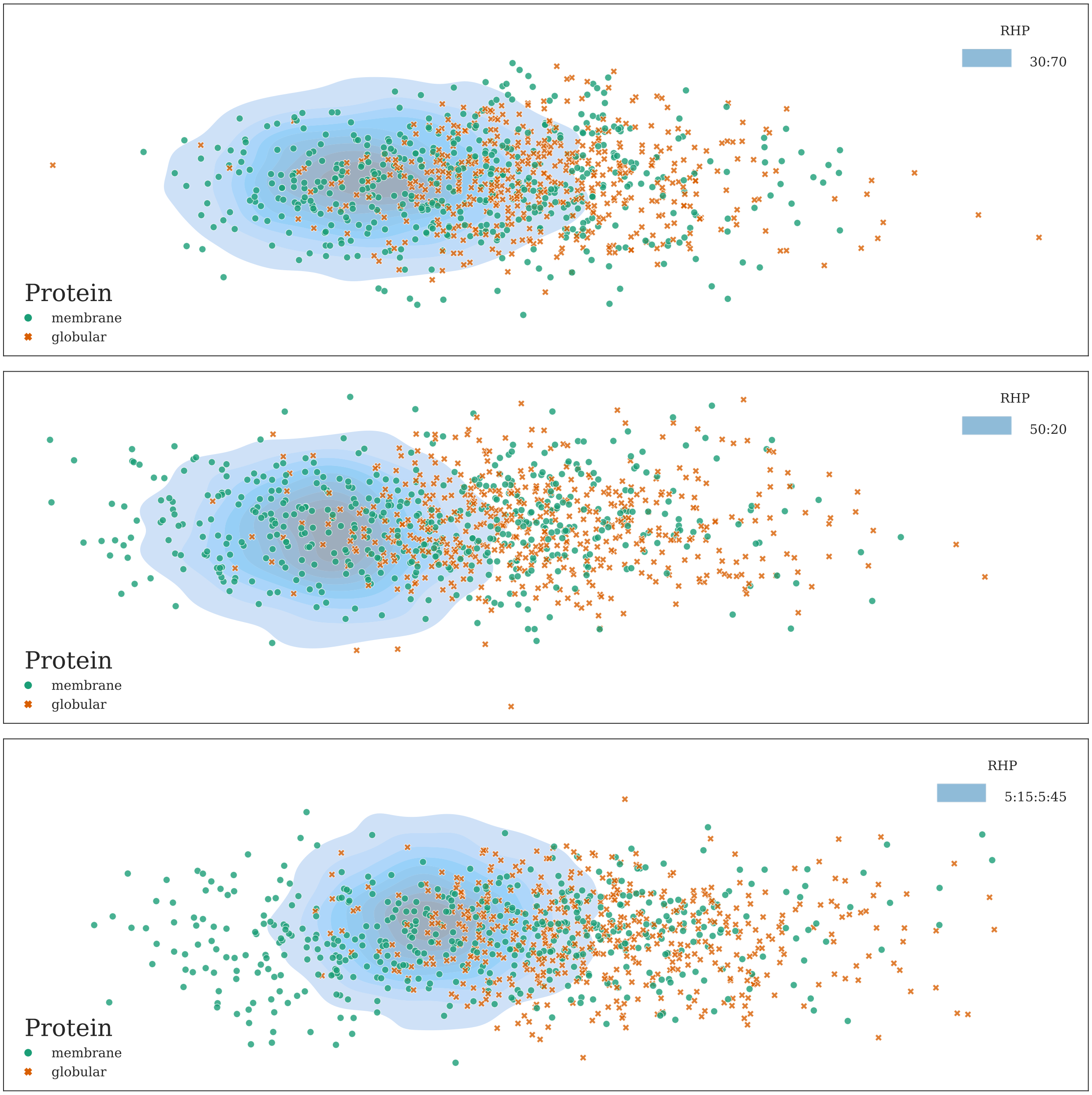


**Fig. S5** The segment distribution of each RHP ensemble and proteins using 2, 4, and 8 pseudo-residues.

(Top) 30:70 is the simulated RHP sequence ensemble composed of 30% EHMA and 70% OEGMA (Mn = 500). (Middle) 50:20 is the simulated sequence ensemble composed of 50% MMA, 20% EHMA, 25% OEGMA (Mn = 500), and 5% SPMA. (Bottom) 5:15:5:45 is the simulated sequence ensemble composed of 5% EHMA, 15% HMA, 5% BMA, 45% MMA, 12.5% OEGMA (Mn = 500), 12.5% OEGMA (Mn = 300), 2.5 % DMAEMA, and 2.5% SPMA. The same protein sequences were analyzed. While the overall molar ratio of hydrophobic/hydrophilic monomers of three RHP ensembles remains the same, an RHP ensemble using more monomers occupied a smaller sequence space compared to the analyzed protein space.

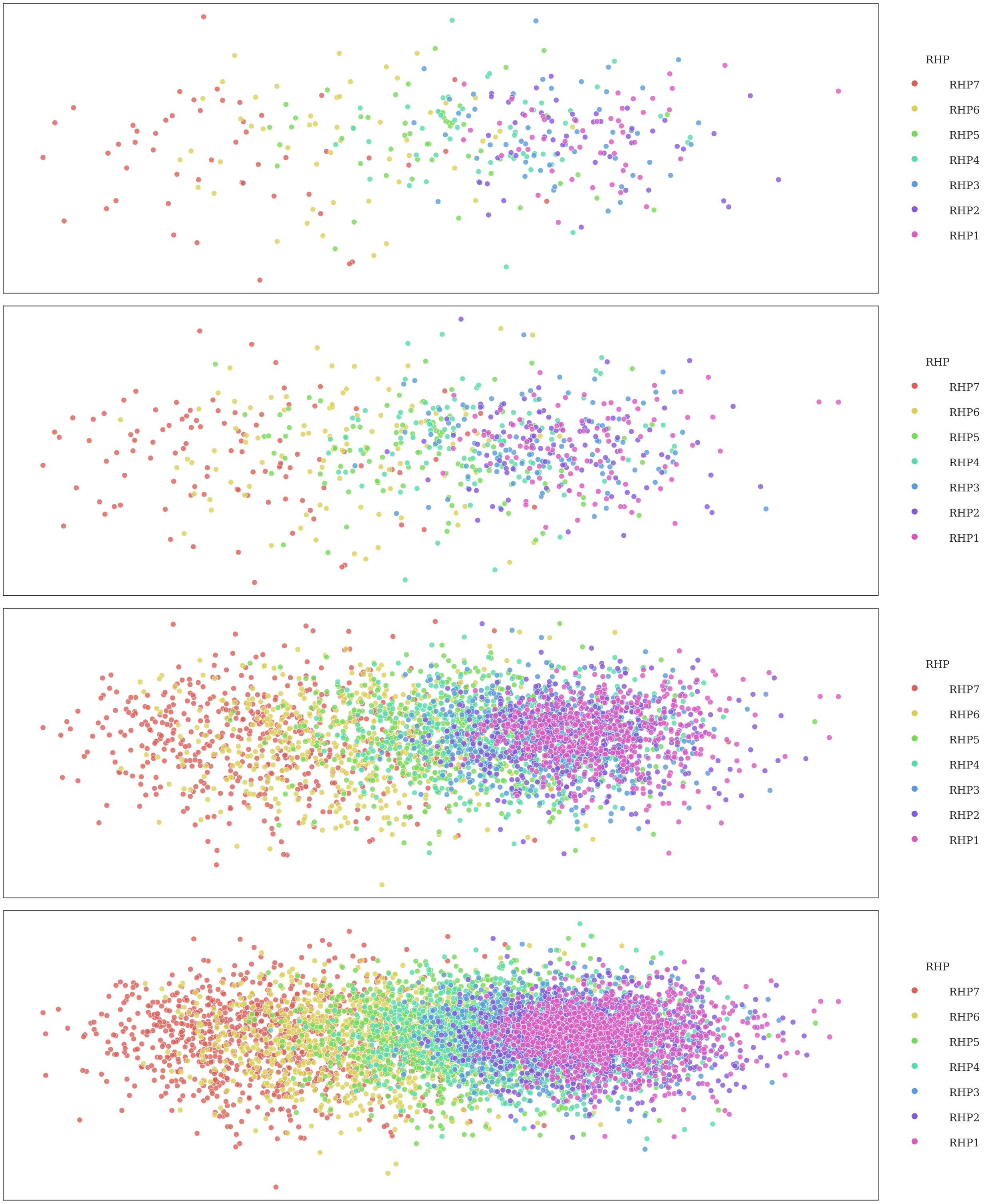
RHP
RHP7
RHP6
RHP5
RHP4
RHP3
RHP2
RHP1

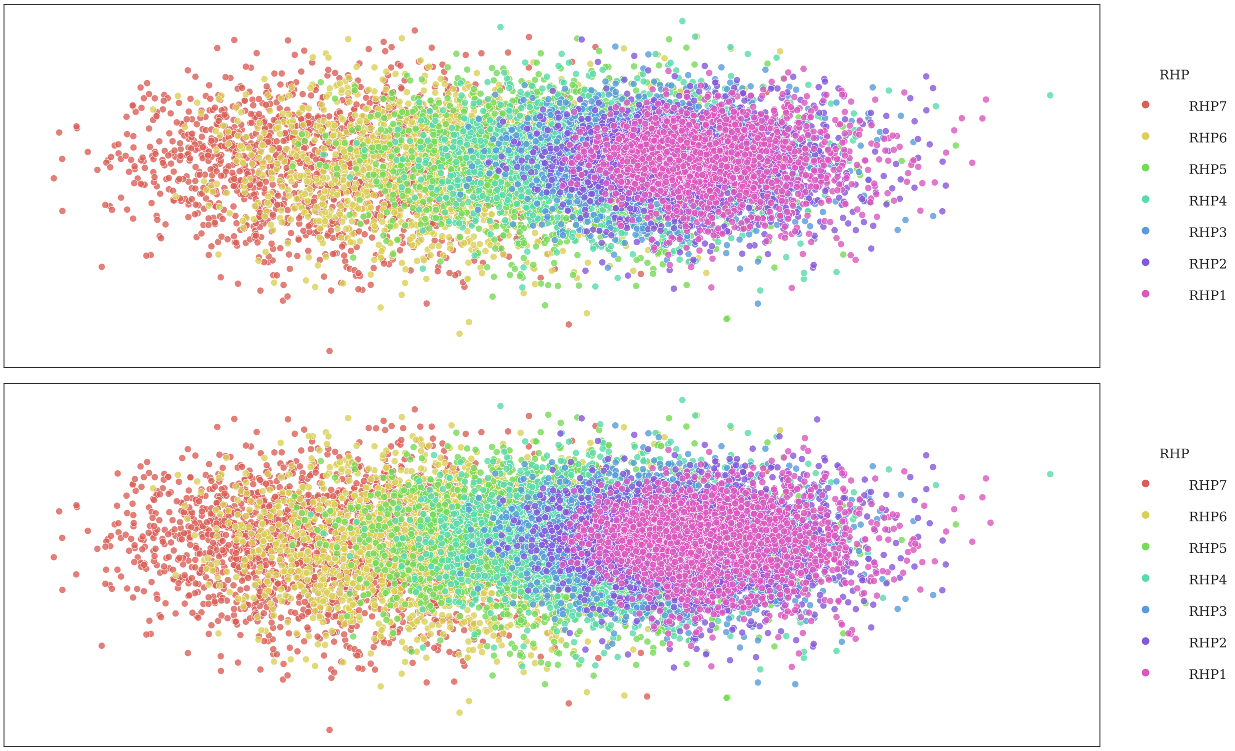


**Fig. S6** The segment distribution of each RHP ensemble as a function of the number of segments (N) per RHP ensemble.

From top to bottom, N is 50, 100, 500, 1000, 1500, and 2000, respectively. Visual inspection of each RHP ensemble shows that the segment distribution converges when N >= 1500 segments per RHP ensemble are analyzed.

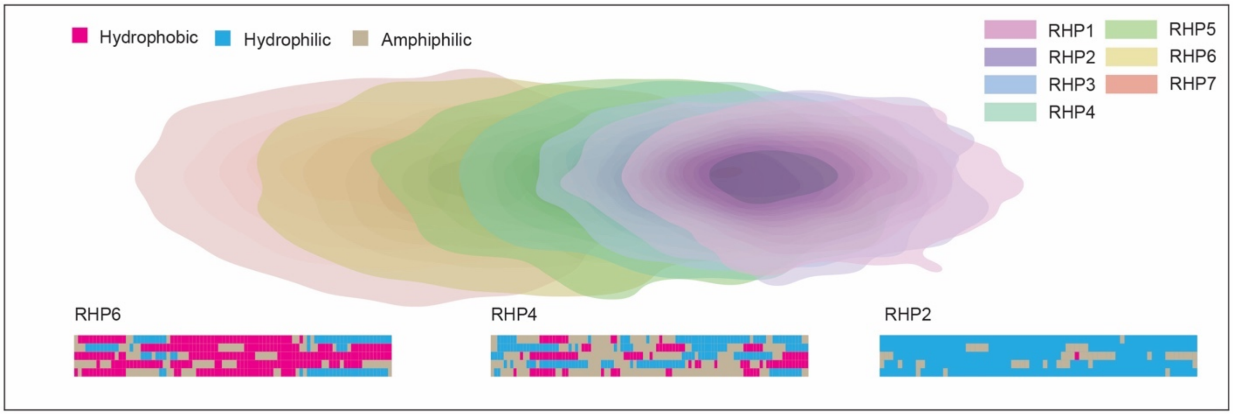


**Fig. S7** The correlation between the relative abundance of different segments and their distribution in PCA space. The hydrophilic, amphiphilic, and hydrophobic segments are highlighted in blue, grey, and pink, respectively. The segments along an RHP chain are shown for three representative RHP ensembles with five individual chains.

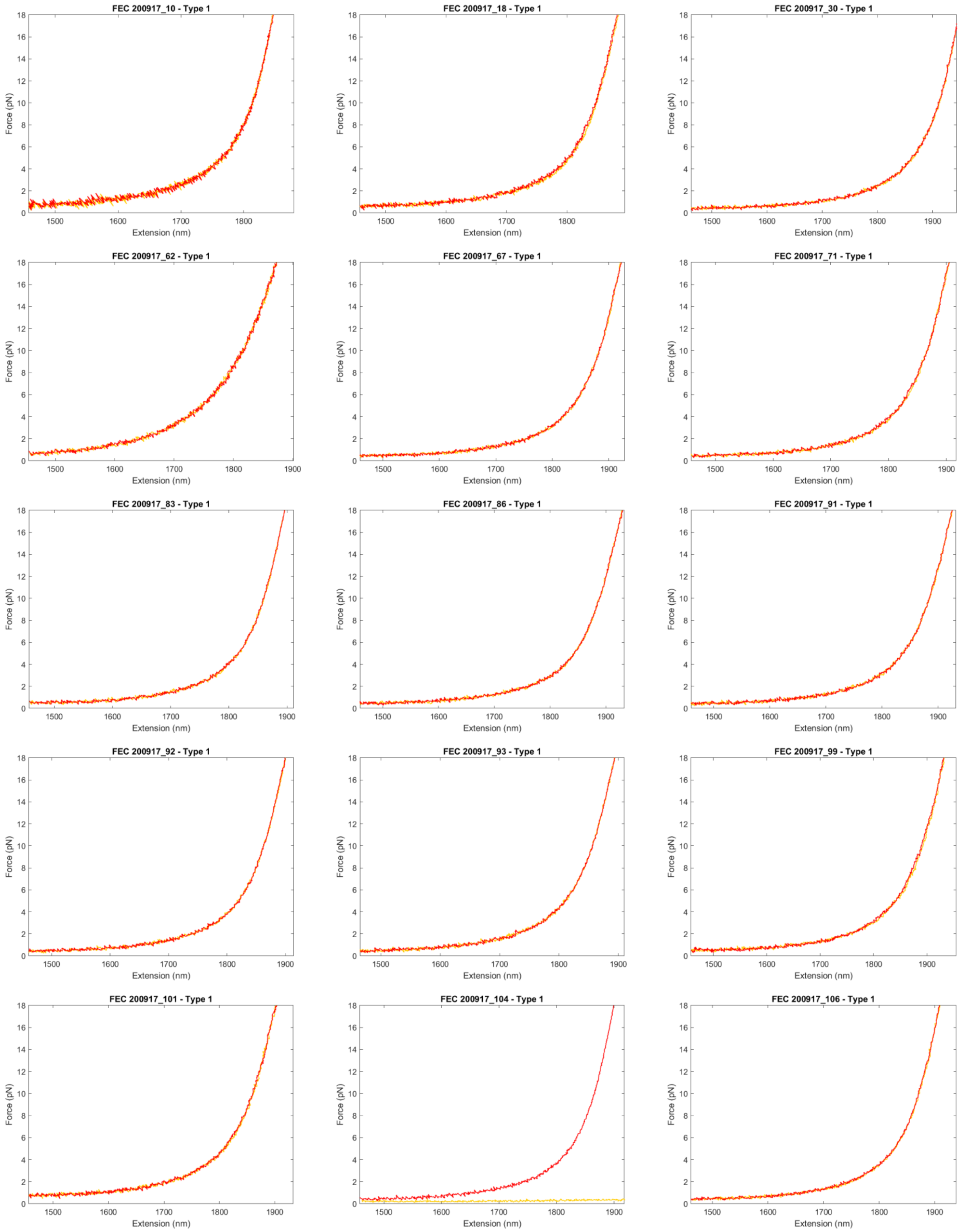
FEC 200917_10 - Type 1
FEC 200917_18 - Type 1
FEC 200917_30 - Type 1
FEC 200917_62 - Type 1
FEC 200917_67 - Type 1
FEC 200917_71 - Type 1
FEC 200917_83 - Type 1
FEC 200917_86 - Type 1
FEC 200917_91 - Type 1
FEC 200917_92 - Type 1
FEC 200917_93 - Type 1
FEC 200917_99 - Type 1
FEC 200917_101 - Type 1
FEC 200917_104 - Type 1
FEC 200917_106 - Type 1
Force (pN)
Extension (nm)

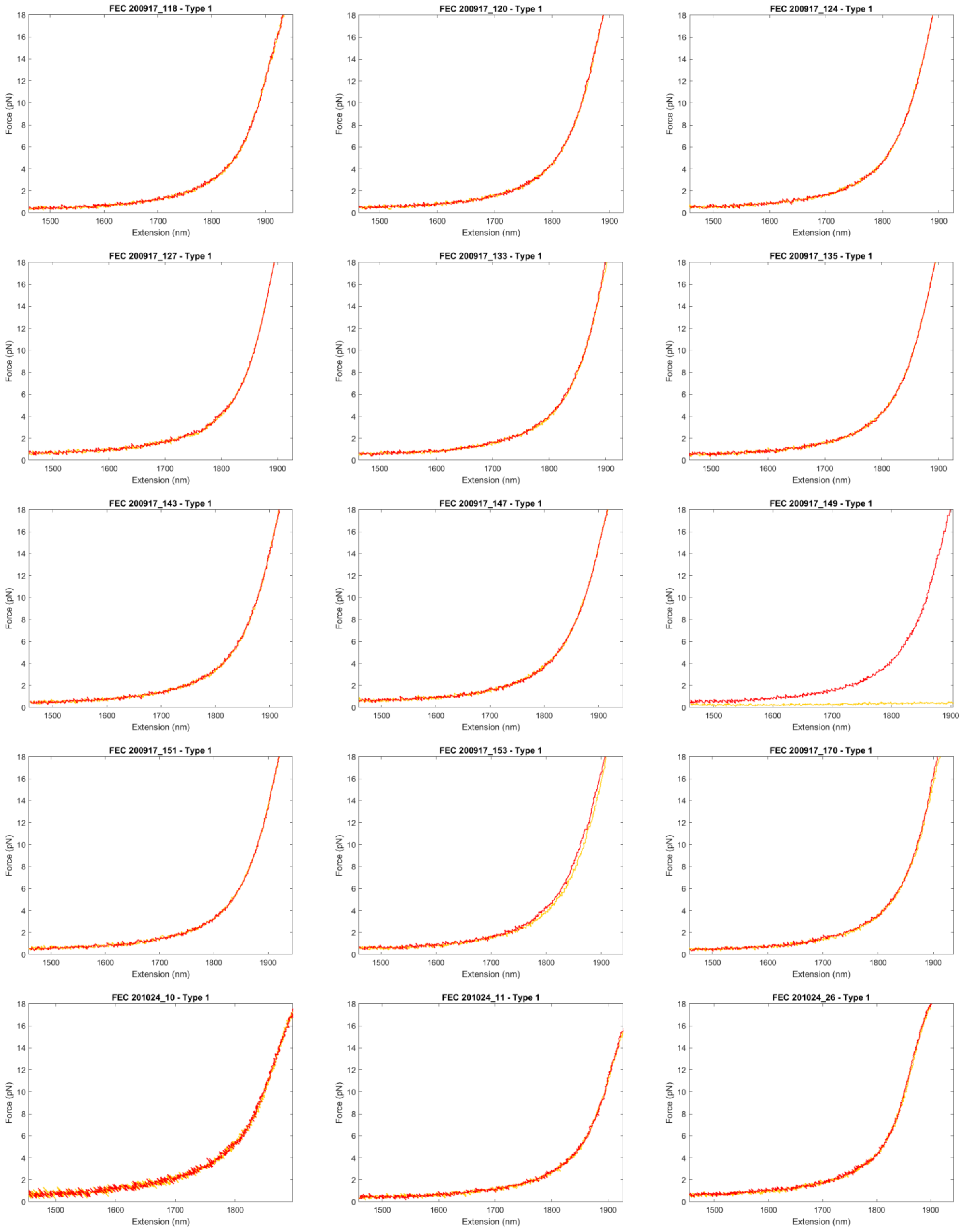
FEC 200917_118 - Type 1
FEC 200917_120 - Type 1
FEC 200917_124 - Type 1
FEC 200917_127 - Type 1
FEC 200917_133 - Type 1
FEC 200917_135 - Type 1
FEC 200917_143 - Type 1
FEC 200917_147 - Type 1
FEC 200917_149 - Type 1
FEC 200917_151 - Type 1
FEC 200917_153 - Type 1
FEC 200917_170 - Type 1
FEC 201024_10 - Type 1
FEC 201024_11 - Type 1
FEC 201024_26 - Type 1
Force (pN)
Extension (nm)

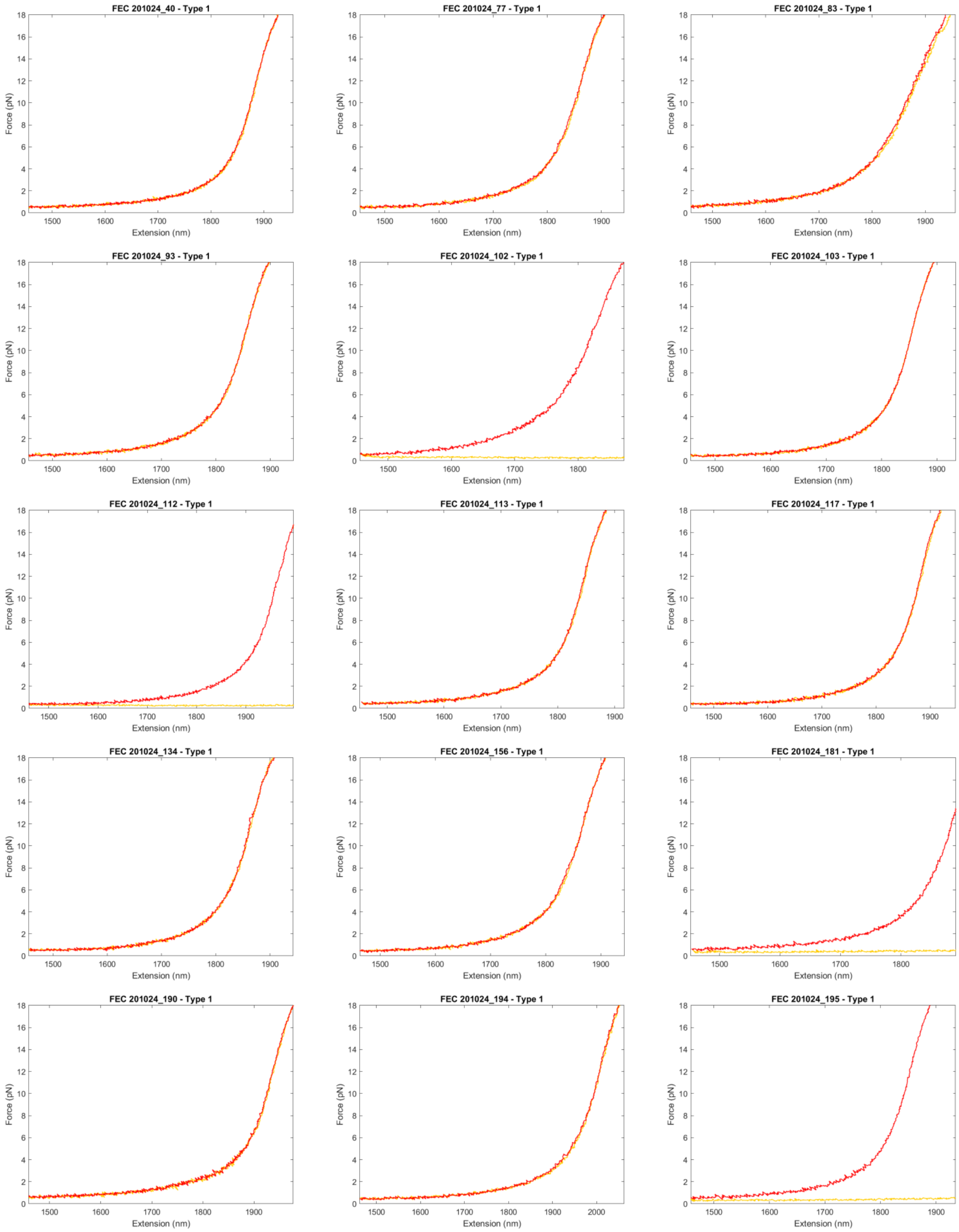
FEC 201024_40 - Type 1
FEC 201024_77 - Type 1
FEC 201024_83 - Type 1
FEC 201024_93 - Type 1
FEC 201024_102 - Type 1
FEC 201024_103 - Type 1
FEC 201024_112 - Type 1
FEC 201024_113 - Type 1
FEC 201024_117 - Type 1
FEC 201024_134 - Type 1
FEC 201024_156 - Type 1
FEC 201024_181 - Type 1
FEC 201024_190 - Type 1
FEC 201024_194 - Type 1
FEC 201024_195 - Type 1
Force (pN)
Extension (nm)

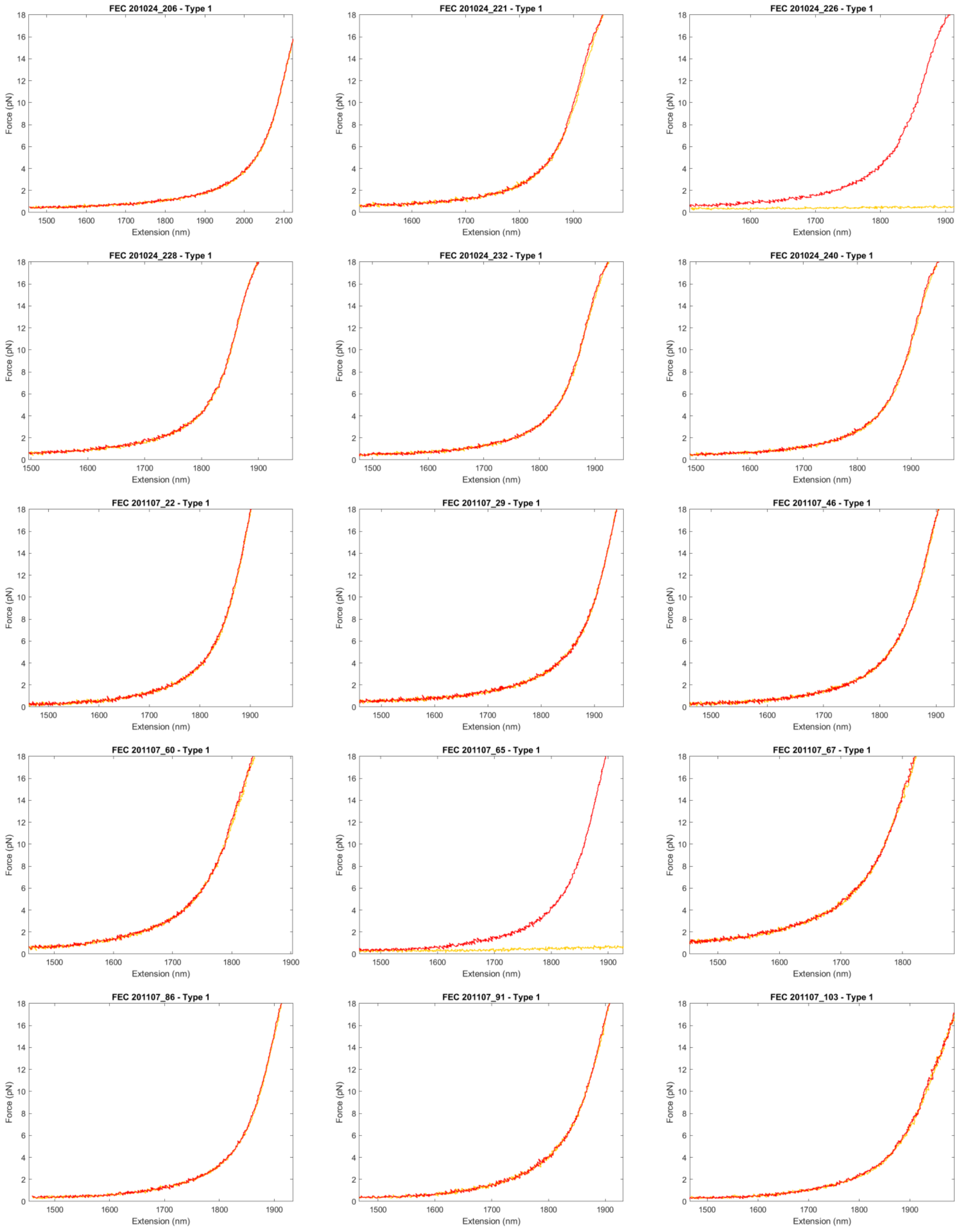
FEC 201024_206 - Type 1
FEC 201024_221 - Type 1
FEC 201024_226 - Type 1
FEC 201024_228 - Type 1
FEC 201024_232 - Type 1
FEC 201024_240 - Type 1
FEC 201107_22 - Type 1
FEC 201107_29 - Type 1
FEC 201107_46 - Type 1
FEC 201107_60 - Type 1
FEC 201107_65 - Type 1
FEC 201107_67 - Type 1
FEC 201107_86 - Type 1
FEC 201107_91 - Type 1
FEC 201107_103 - Type 1
Force (pN)
Extension (nm)

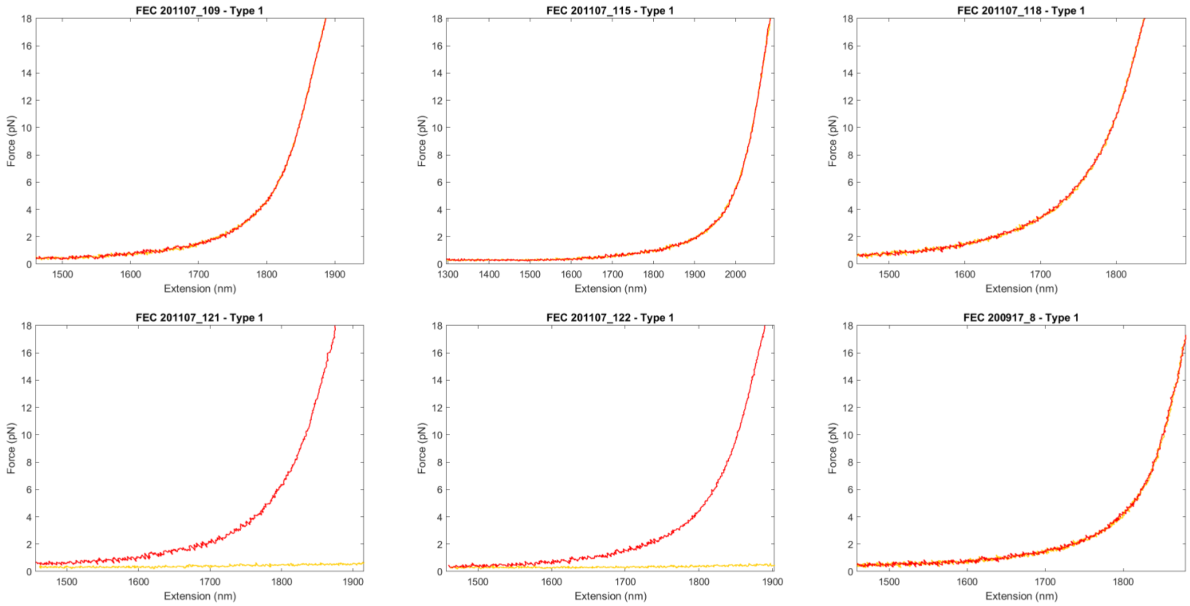


**Fig. S8** All FEC traces upon pulling (red) and relaxing (orange) showing no unfolding signature. A flat relaxing curve indicates that the tether breaks before relaxation.

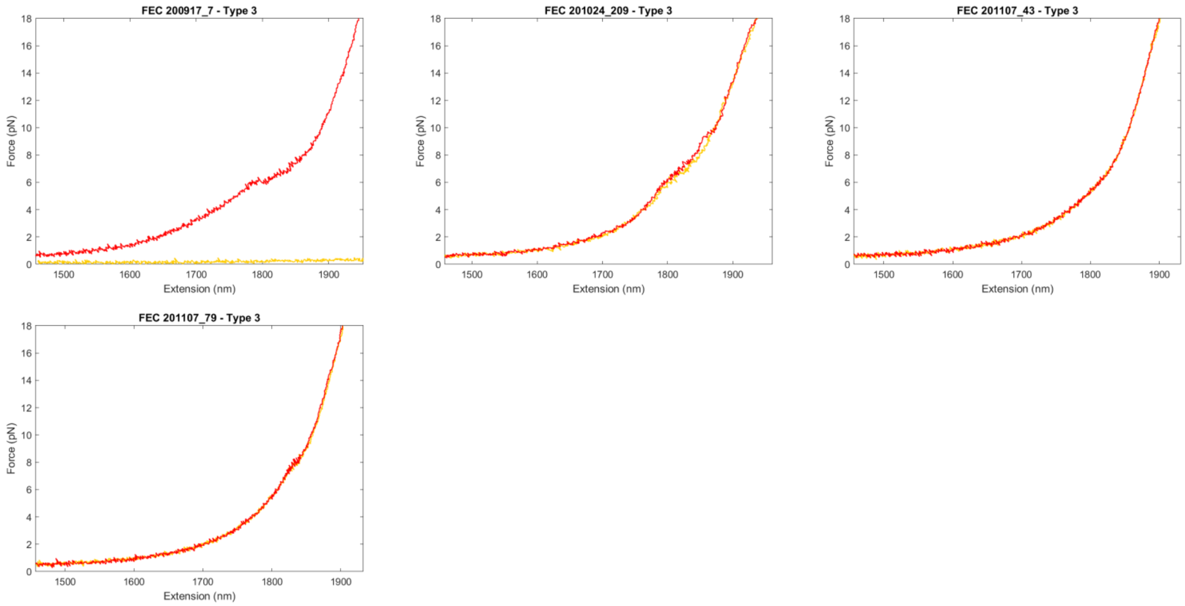


**Fig. S9** All FEC traces upon pulling (red) and relaxing (orange) showing smooth shoulders. A flat relaxing curve indicates that the tether breaks before relaxation.

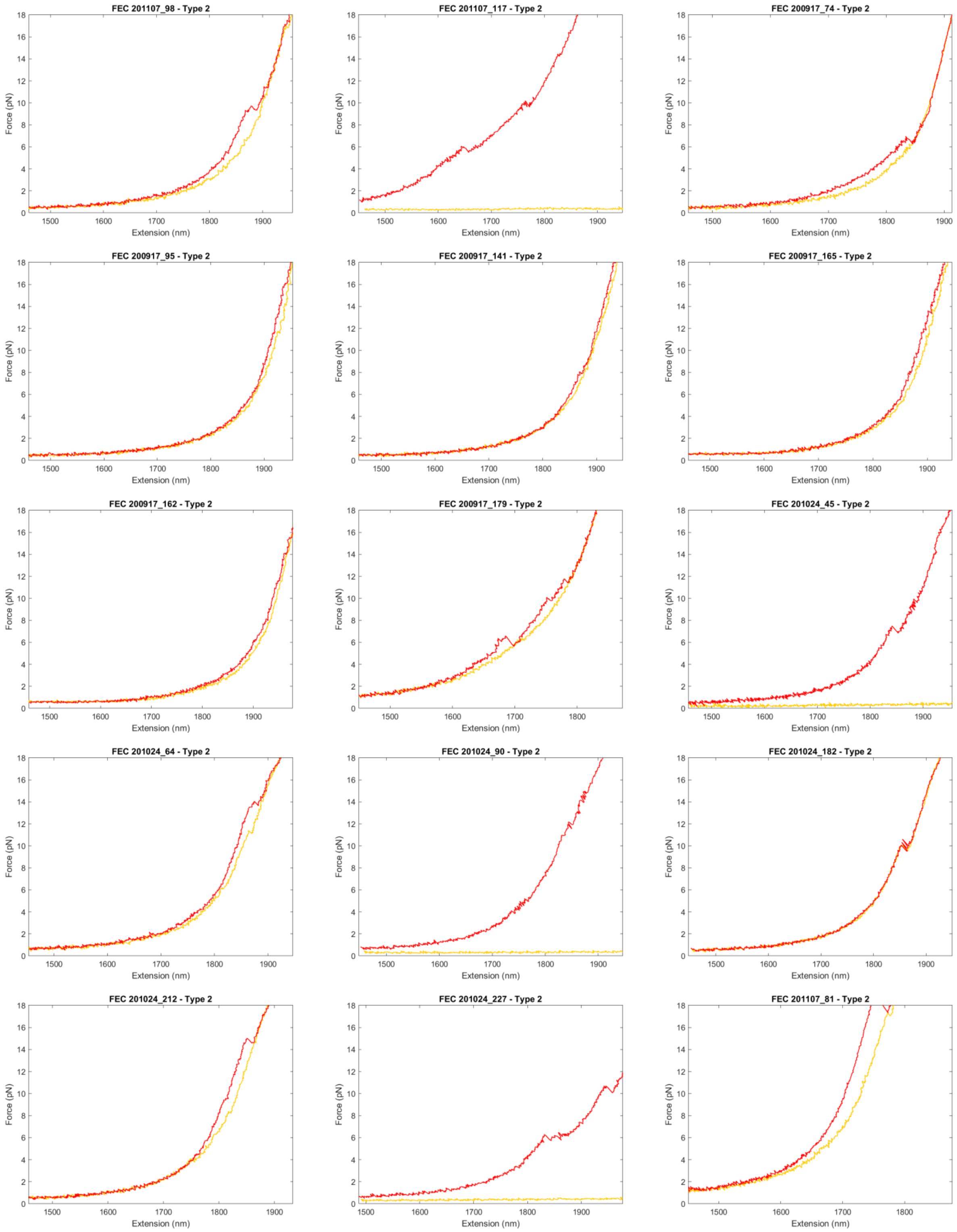


**Fig. S10** All FEC traces upon pulling (red) and relaxing (orange) showing discrete rips. A flat relaxing curve indicates that the tether breaks before relaxation.

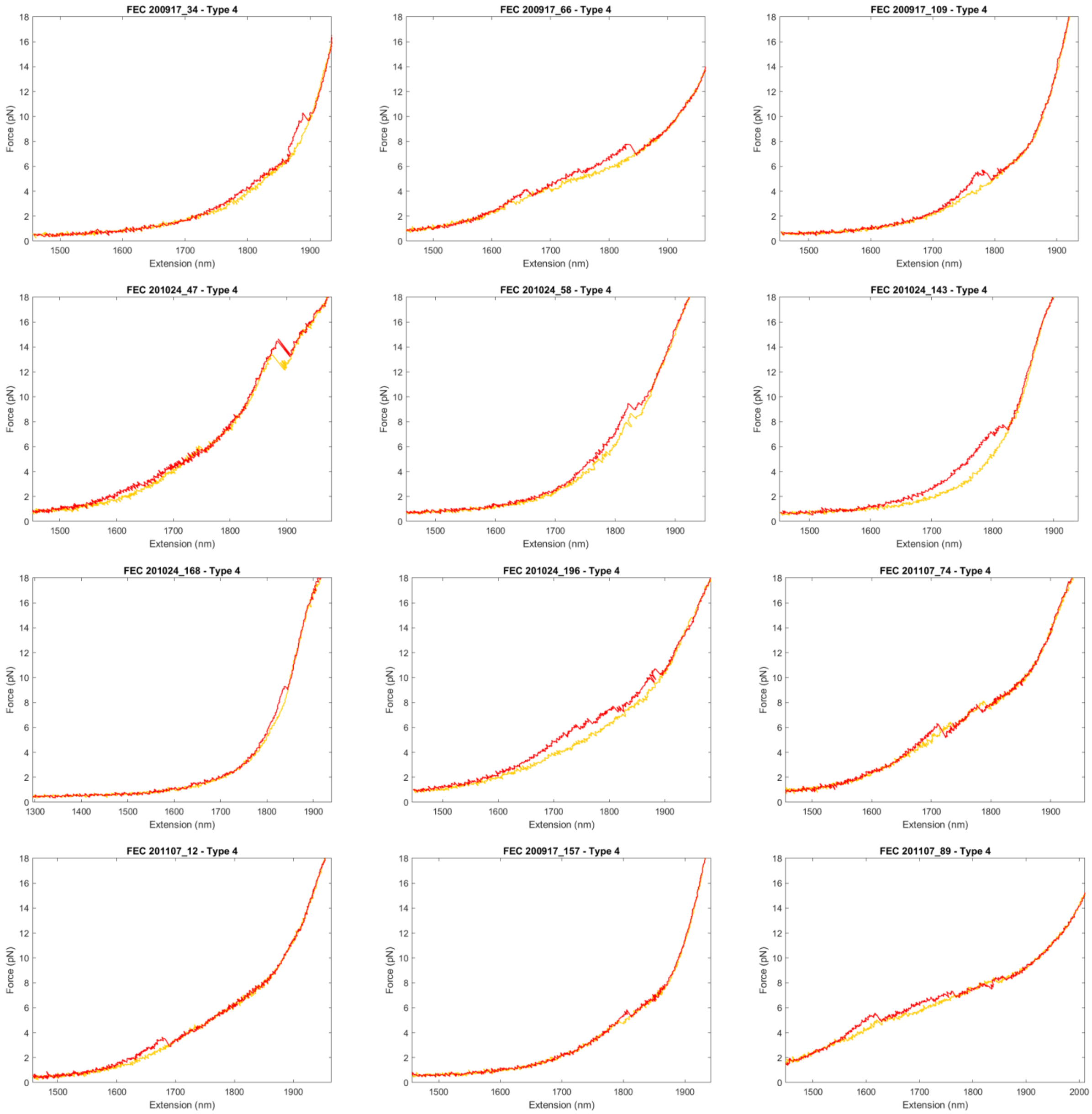


**Fig. S11** All FEC traces upon pulling (red) and relaxing (orange) showing a mixture of rips and shoulders. A flat relaxing curve indicates that the tether breaks before relaxation.

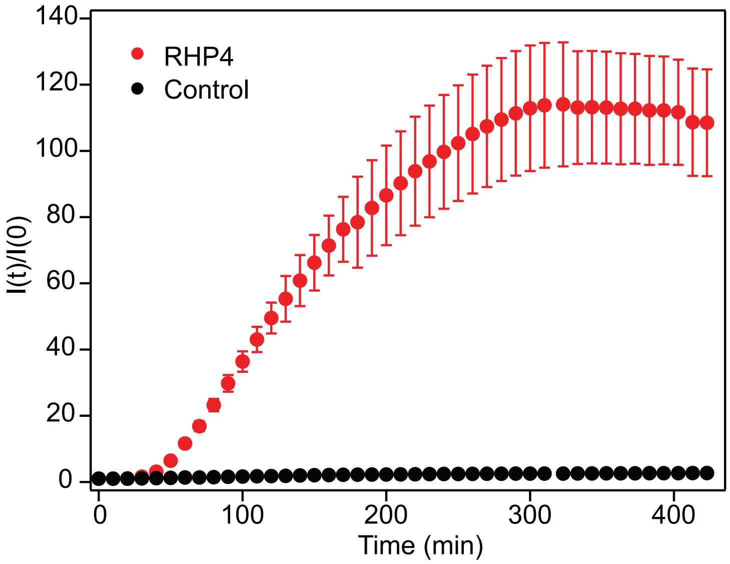


**Fig. S12** AqpZ-eGFP fluorescence in the presence/absence of RHP ensembles during cell-free expression. Error bar is 1 s.d. (n=3).

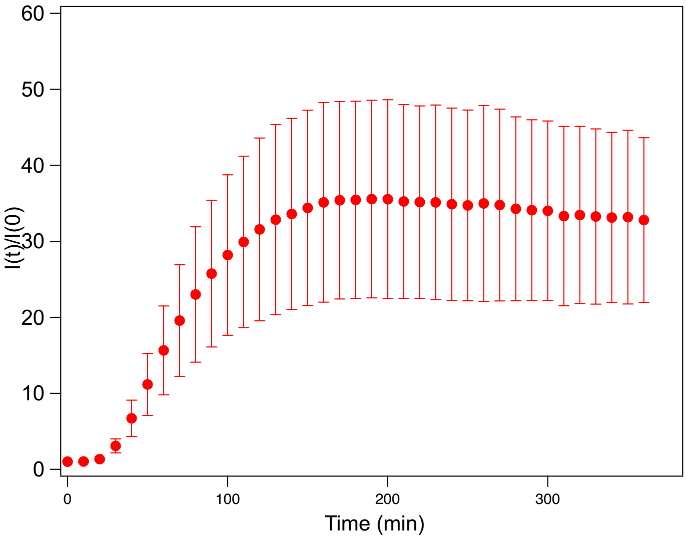


**Fig. S13** OmpT-eGFP fluorescence in the absence of RHP ensembles during cell-free expression (n=3). Error bar is 1 s.d.

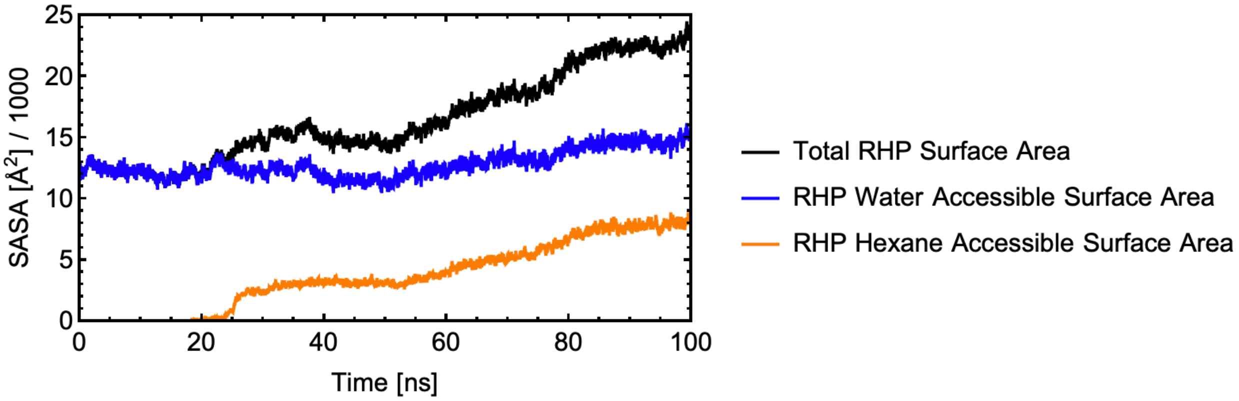


**Fig. S14** The solvent accessible surface area (SASA) of a representative RHP4 particle over 100 ns of MD simulation.

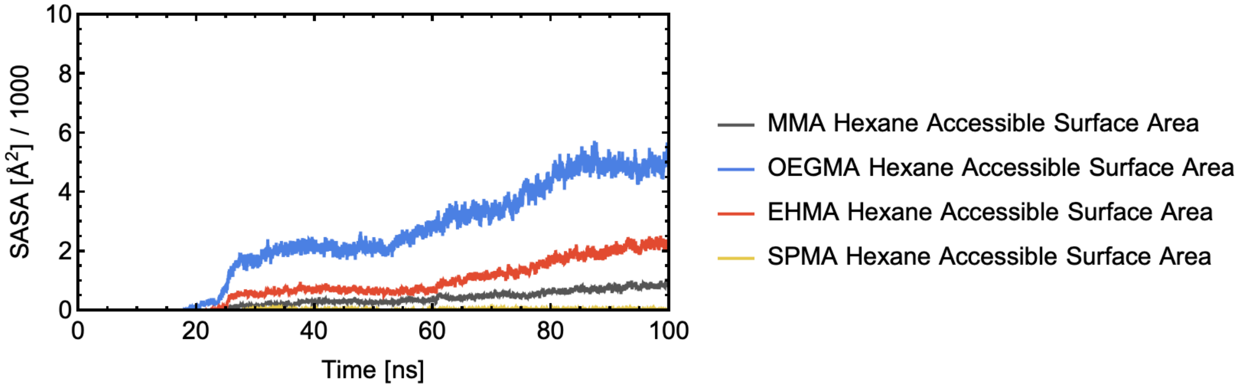


**Fig. S15** The solvent accessible surface area (SASA) of each type of monomer toward the hexane phase over 100 ns of MD simulation.

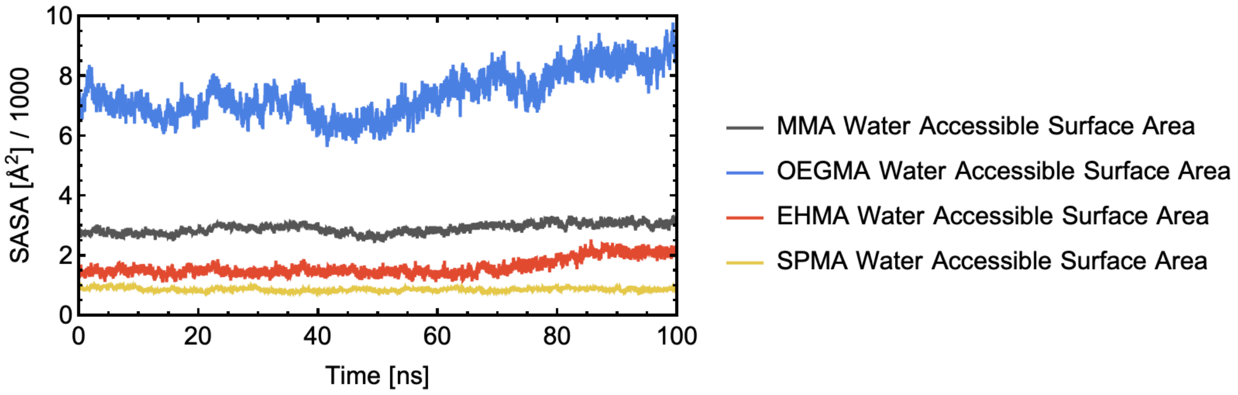


**Fig. S16** The solvent accessible surface area (SASA) of each type of monomer toward the water phase over 100 ns of MD simulation.

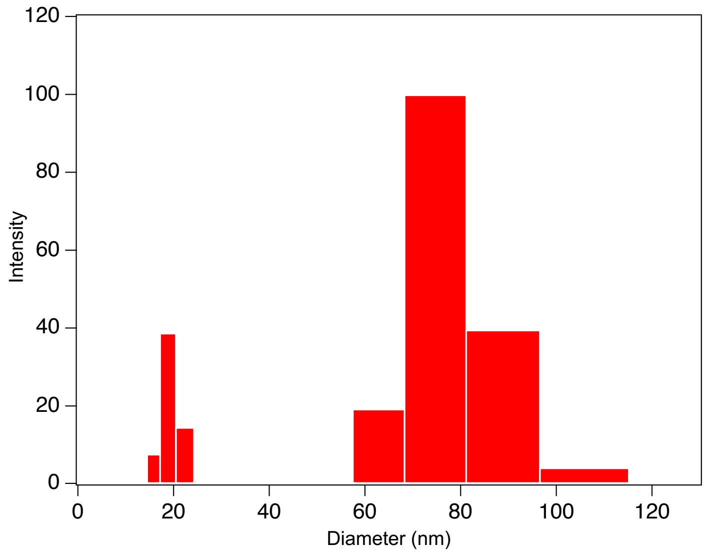


**Fig. S17** DLS measurement of DHP1 polymer solution.

DHP1 showed a bimodal size distribution by the intensity with a mean diameter of 56.2 nm (CONTIN). This result suggests that DHP1 forms large assemblies in water.

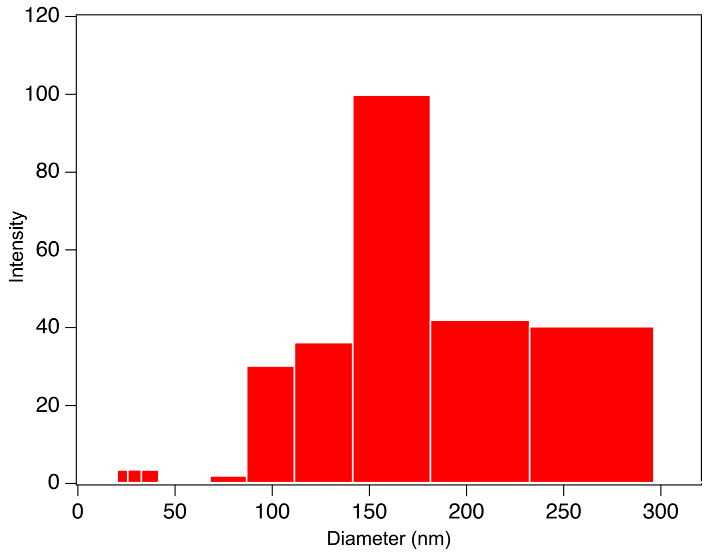


**Fig. S18** DLS measurement of DHP2 polymer solution.

DHP2 showed bimodal size distribution by the intensity with a mean diameter of 145.5 nm (CONTIN). This result suggests that DHP2 forms large assemblies in water.

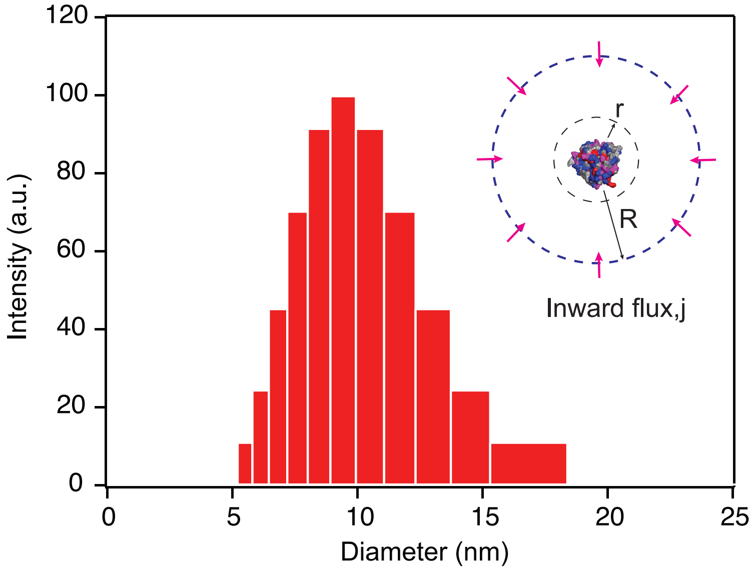


**Fig. S19** DLS measurement of RHP4 polymer solution.

RHP4 showed unimodal size distribution by the intensity with a mean diameter of 9.1 nm (CONTIN) , corresponding to a diffusion coefficient (D) of 54 μm$^2$/s (in water).

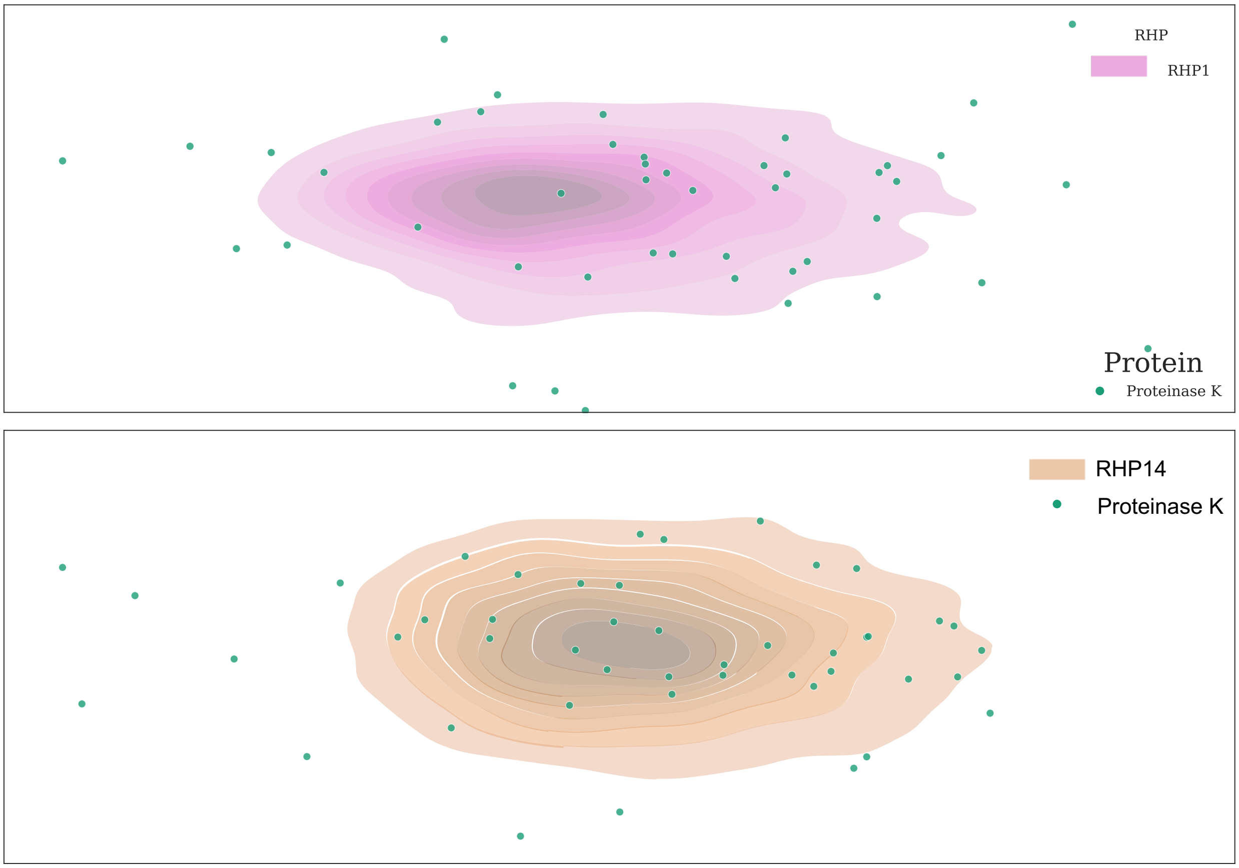


**Fig. S20** The segment distribution of RHP1, RHP14 and Proteinase K in PCA space.

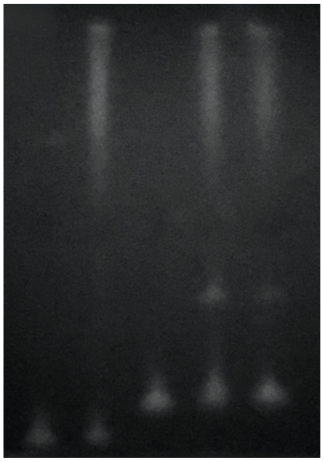

**Fig. S21** 8% PAGE analysis showing the formation of DNA-RHP conjugates using thiol-maleimide addition.

Lanes from left to right: DNA2, DNA2-RHP4, DNA1, DNA1-RHP4 (conjugated in a cosolvent of DMF:$H_2O$ = 1:1, vol%), DNA1-RHP4 (conjugated in a cosolvent of DMF:$H_2O$ = 1:9, vol%). The smear bands on the top are the DNA-RHP4 conjugates.
